# Targeted Antimicrobial Photodynamic Therapy of Biofilm-Embedded and Intracellular Staphylococci with a Phage Endolysin’s Cell Binding Domain

**DOI:** 10.1128/spectrum.01466-21

**Published:** 2022-02-23

**Authors:** Mafalda Bispo, Sílvio B. Santos, Luís D. R. Melo, Joana Azeredo, Jan Maarten van Dijl

**Affiliations:** a Department of Medical Microbiology, University of Groningen, University Medical Center Groningen, Groningen, the Netherlands; b Centre of Biological Engineering, University of Minho, Braga, Portugal; Riverside University Health System, Medical Center, University of California

**Keywords:** cell-binding domain, endolysin, antimicrobial photodynamic therapy, intracellular *Staphylococcus aureus*, *Staphylococcus epidermidis*, *Staphylococcus aureus*, biofilms, intracellular bacteria

## Abstract

Bacterial pathogens are progressively adapting to current antimicrobial therapies with severe consequences for patients and global health care systems. This is critically underscored by the rise of methicillin resistant Staphylococcus aureus (MRSA) and other biofilm-forming staphylococci. Accordingly, alternative strategies have been explored to fight such highly multidrug resistant microorganisms, including antimicrobial photodynamic therapy (aPDT) and phage therapy. aPDT has the great advantage that it does not elicit resistance, while phage therapy allows targeting of specific pathogens. In the present study, we aimed to merge these benefits by conjugating the cell-binding domain (CBD3) of a Staphylococcus aureus phage endolysin to a photoactivatable silicon phthalocyanine (IRDye 700DX) for the development of a Staphylococcus-targeted aPDT approach. We show that, upon red-light activation, the resulting CBD3-700DX conjugate generates reactive oxygen species that effectively kill high loads of planktonic and biofilm-resident staphylococci, including MRSA. Furthermore, CBD3-700DX is readily internalized by mammalian cells, where it allows the targeted killing of intracellular MRSA upon photoactivation. Intriguingly, aPDT with CBD3-700DX also affects mammalian cells with internalized MRSA, but it has no detectable side effects on uninfected cells. Altogether, we conclude that CBD3 represents an attractive targeting agent for Staphylococcus-specific aPDT, irrespective of planktonic, biofilm-embedded, or intracellular states of the bacterium.

**IMPORTANCE** Antimicrobial resistance is among the biggest threats to mankind today. There are two alternative antimicrobial therapies that may help to control multidrug-resistant bacteria. In phage therapy, natural antagonists of bacteria, lytic phages, are harnessed to fight pathogens. In antimicrobial photodynamic therapy (aPDT), a photosensitizer, molecular oxygen, and light are used to produce reactive oxygen species (ROS) that inflict lethal damage on pathogens. Since aPDT destroys multiple essential components in targeted pathogens, aPDT resistance is unlikely. However, the challenge in aPDT is to maximize target specificity and minimize collateral oxidative damage to host cells. We now present an antimicrobial approach that combines the best features of both alternative therapies, namely, the high target specificity of phages and the efficacy of aPDT. This is achieved by conjugating the specific cell-binding domain from a phage protein to a near-infrared photosensitizer. aPDT with the resulting conjugate shows high target specificity toward MRSA with minimal side effects.

## INTRODUCTION

Antimicrobial resistance is rapidly rising to worrisome levels all over the world, leading to increased mortality and morbidity rates, longer hospital stays, and high economic losses (1). This focuses attention on Staphylococcus aureus, one of the most prominent drug-resistant bacterial pathogens. In particular, methicillin-resistant S. aureus (MRSA) emerged more than 60 years ago and rapidly became a dominant threat to health care on a global scale (2). One factor that strongly contributes to antimicrobial resistance is the ability of S. aureus to form thick biofilms on tissues and implanted biomaterials, which protects the bacteria from the host immune defenses and antimicrobial agents (3). Moreover, S. aureus is a facultative intracellular pathogen that is even capable of using immune cells, such as macrophages and neutrophils, as “Trojan horses” for dissemination throughout the human body (4–6). Altogether, upon host invasion, this pathogen can overcome all innate and adaptive immune defenses, making antimicrobial therapy inevitable (7). Thus, it remains critical to develop novel alternative strategies that allow us to eliminate also the most drug resistant lineages of S. aureus.

Antimicrobial photodynamic therapy (aPDT) is a promising alternative strategy for tackling antimicrobial resistance. With no bacterial resistance reported so far (8–10), this therapeutic approach is based on the combination of three essential elements: light, molecular oxygen, and a photosensitizer. Light of a specific wavelength will photoactivate the photosensitizer by exciting it to a triple-excited state, which will then react with molecular oxygen to form reactive oxygen species (ROS) that will, in turn, kill the bacteria (11). Ideally, the photosensitizer should be coupled to a targeting agent to achieve higher specificity and efficacy. We previously developed an immunoconjugate based on a S. aureus-specific human monoclonal antibody and the photosensitizer IRDye 700DX (12). This immunoconjugate was capable of killing planktonic and biofilm-embedded S. aureus, showing *in vivo* efficacy with minimal cytotoxic side effects toward mammalian cells (12). However, the Achilles heel of monoclonal antibodies is that they address only one specific target epitope. Such targets may mutate, and this could eventually lead to resistance, rendering an antibody-based drug inactive. Therefore, it is important to develop other conjugates which preferentially target conserved and stable regions in S. aureus cells for use in aPDT approaches.

Phage therapy started to emerge about a century ago as an alternative antimicrobial strategy (13). Due to their high species-specificity, bacteriophages are also potential candidates to target photosensitizers for aPDT. Indeed, there are a few reports of photosensitizers conjugated to bacteriophages to treat bacterial infections (14, 15). Lytic bacteriophages interact with receptors on the bacterial cell surface to inject their genome into the bacterium, which is then followed by the production of viral progeny. To release this progeny into the environment, holins and endolysins are produced, which create pores in the cytoplasmic membrane and hydrolyze the surrounding cell envelope, respectively (13). Nevertheless, phage therapy also suffers from the development of bacterial resistance (16). Therefore, the possibility of making use of the lytic activity of phage endolysins was explored. Such endolysins can be applied exogenously, making them appealing candidates for antimicrobial treatment (17–19). Endolysins derived from phages that infect staphylococci usually have two catalytic domains for enzymatic activity: an N-terminal, cysteine, histidine-dependent amidohydrolase/peptidase domain, and a central *N*-acetylmuramoyl-l-alanine amidase domain (Ami). In addition, these enzymes have a cell-binding domain (CBD) at the C terminus (20, 21). CBDs are responsible for the high specificity of endolysins for Gram-positive bacteria, and they usually display higher target-binding affinity than antibodies, as well as high stability to temperature and pH variations. Accordingly, it was previously shown that CBDs may serve as promising probes for the rapid detection of bacterial infections (22, 23). For instance, Costa et al. have truncated the S. aureus phage endolysin E-LM12, producing a molecule composed of the Ami domain plus the C-terminal CBD, which belongs to the ‘SRC Homology 3b’ (SH3b) class that binds staphylococcal peptidoglycan cross-bridges. This truncated version of the endolysin, which was named CBD3 (formerly, also referred to as AMI_SH3), allows the rapid detection of S. aureus in blood cultures (23).

This study was aimed at investigating whether CBD3 could serve as a targeting agent for aPDT. Here, we report the successful conjugation of CBD3 with the photosensitizer IRDye 700DX and the subsequent application of the resulting CBD3-700DX conjugate in aPDT approaches to eliminate staphylococci *in vitro*, in biofilms, and inside human host cells. In fact, our study represents the first usage of a truncated endolysin as a targeting agent for aPDT.

## RESULTS

### CBD3 binding to Staphylococcus species.

**FIG 1:**
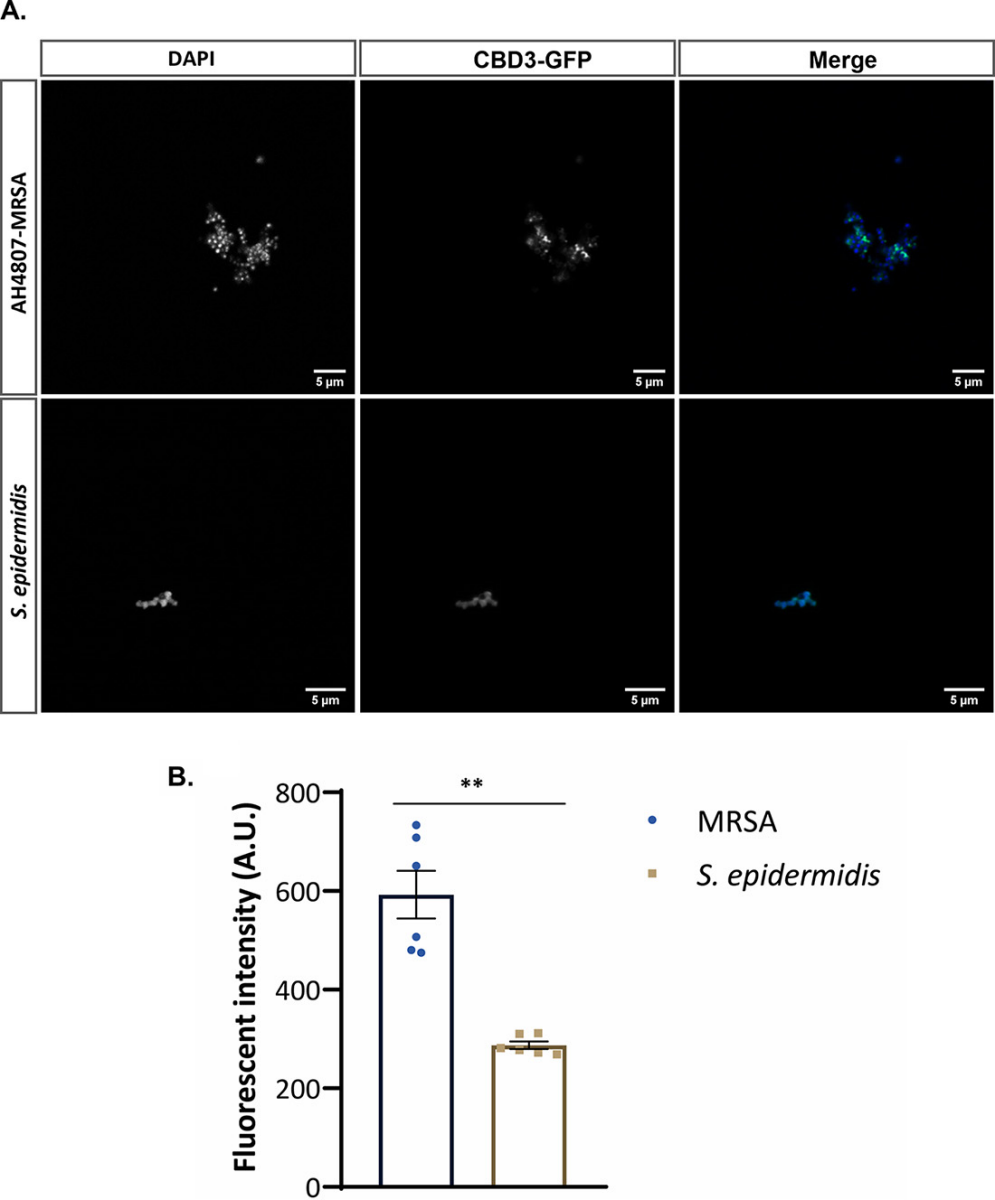
(A) Co-localization of the GFP-CBD3 fusion protein (green) with bacteria of the CA-MRSA strain AH4807 and the S. epidermidis ATCC 35984 strain stained with DAPI (blue). A total of 1 μM GFP-CBD3 was incubated with diluted bacterial overnight cultures (OD_600_ = 0.5) and imaged by confocal laser scanning microscopy. (B) Binding intensity of 0.64 μM CBD3-700DX to bacteria of the CA-MRSA-AH4807 strain and the S. epidermidis ATCC strain 35984. Fluorescence was measured with an Amersham Typhoon Biomolecular Imager and analyzed with ImageJ. Data are presented as the mean ± standard error of the mean (SEM) of two experiments performed in triplicate. Welch’s test was used for statistical analysis. Significant differences are marked (**, *P* < 0.002).

To validate our experimental setup for CBD3-based aPDT, we first evaluated the binding of a fusion protein, consisting of green fluorescent protein (GFP) fused to the N terminus of CBD3 (GFP-CBD3), to two representative Staphylococcus species, namely, S. aureus and Staphylococcus epidermidis. In particular, we applied two model strains, one of each species: namely, the bioluminescent MRSA strain AH4807, which belongs to the community-acquired (CA) USA300 lineage of S. aureus; and the S. epidermidis strain ATCC 35984, which is known to form thick biofilms. As a first approach, binding of GFP-CBD3 to these two bacterial strains was evaluated by confocal scanning fluorescence microscopy (Fig. 1). As expected, GFP-CBD3 allowed rapid detection of the two strains, and the GFP signal co-localized with the signal of 4′,6-diamidino-2-phenylindole (DAPI) bound to the bacterial DNA (Fig. 1A). Subsequently, CBD3 was conjugated with IRDye 700DX, and the binding of the resulting CBD3-700DX to the two staphylococcal strains was quantified by fluorescence imaging. As shown in Fig. 1B, the binding of CBD3-700DX to the MRSA bacteria was significantly more efficient than the binding to the S. epidermidis bacteria, resulting in a higher fluorescence intensity for the MRSA cells. Although the precise binding site of CBD3 in the bacterial cell wall has not yet been determined, it is known from literature that SH3b domains usually bind the pentaglycine cross-bridges in staphylococcal peptidoglycan (24). While S. aureus cell walls contain a high proportion of pentaglycine cross-bridges, the coagulase-negative staphylococci, including S. epidermidis, also incorporate amino acids other than glycine (predominantly serine and alanine) in their peptidoglycan cross-bridges, which would reduce the binding of SH3b domains (25). This would explain the lower binding of CBD3-700DX to the S. epidermidis bacteria compared to the MRSA bacteria.

### Effective killing of MRSA and *S. epidermidis* by aPDT with CBD3-700DX.

**FIG 2:**
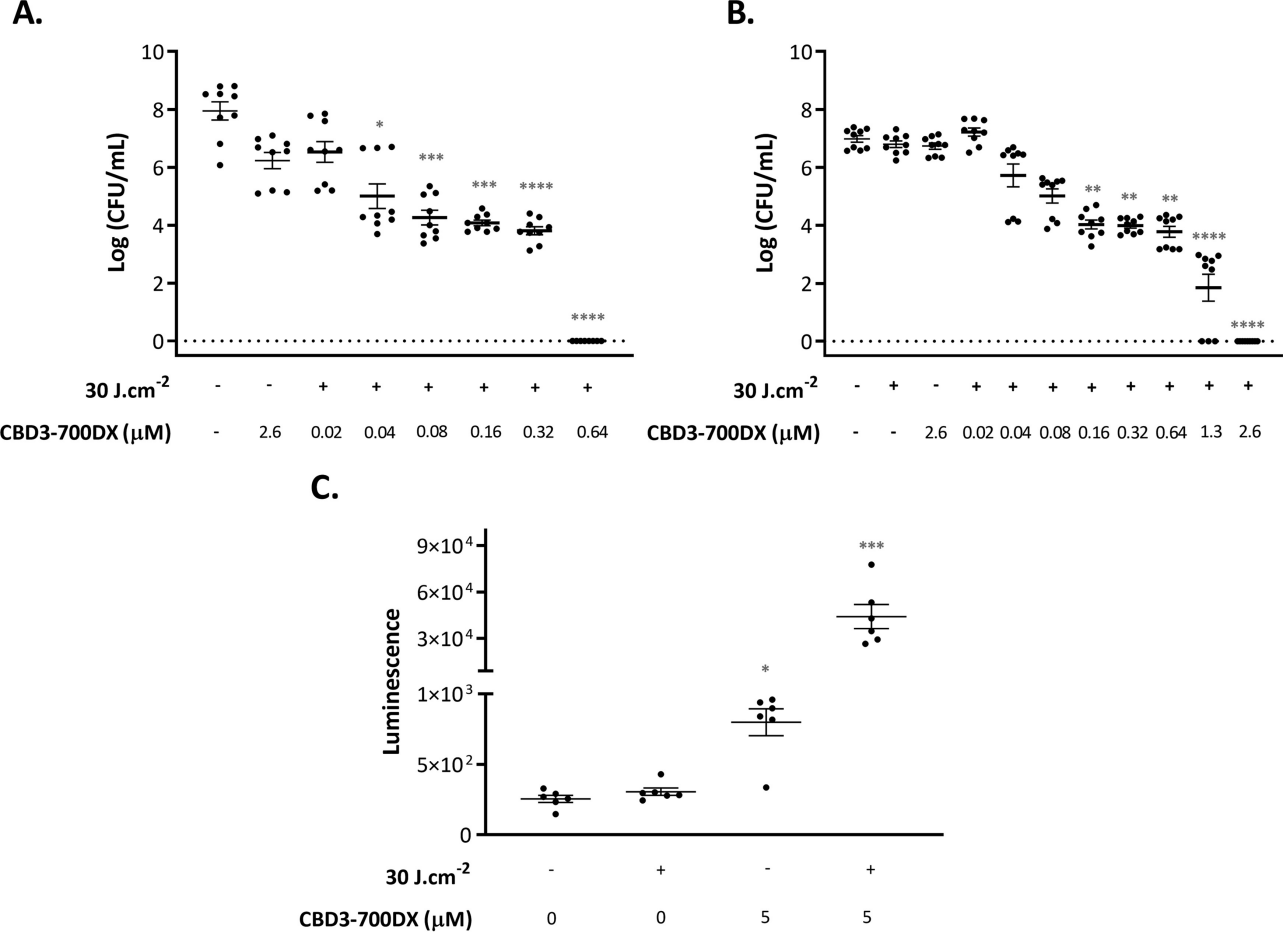
Photoactivated killing of S. aureus CA-MRSA-AH4807 (A) and S. epidermidis ATCC 35984 (B) grown to exponential phase. Approximately 1 × 10^7^ CFU/mL of the bacteria were incubated with stepwise increasing concentrations of CBD3-700DX (0.02 to 2.6 μM) or without photosensitizer. Bacteria were irradiated with red light at a radiance exposure of 30 J · cm^−2^ (+) or kept in the dark (–). (C) H_2_O_2_ production upon aPDT of CA-MRSA-AH4807 with 5 μM CBD3-700DX or without photosensitizer. H_2_O_2_ was detected with 10 μM of an AquaSpark Peroxide Probe. In all experiments, irradiation was performed with a LED system that emits red light. Data are presented as the mean ± SEM of three experiments performed in triplicate (panels A and B) and two experiments performed in triplicate (panel C). The Kruskal-Wallis test with subsequent Dunn’s multiple-comparison tests was used for statistical analysis. Significant differences compared with the negative-control group (no CBD3-700DX and no light) are shown as follows: *, *P* < 0.03; **, *P* < 0.002; ***, *P* < 0.0002; ****, *P* < 0.0001.

To investigate the antimicrobial efficacy of CBD3-700DX after photoactivation, we assessed bacterial survival in the presence or absence of the conjugate in the dark and after red-light activation (Fig. 2A and B). Approximately 10^7^ CFU/mL of MRSA-AH4807 or S. epidermidis ATCC 35984 were incubated with increasing concentrations of CBD3-700DX. The bacteria were then either kept in the dark or irradiated with a red-light LED system (690 nm) (26) at a radiance exposure of 30 J · cm^−2^. The results show that concentrations of 0.64 and 2.6 μM can fully eradicate MRSA-AH4807 (Fig. 2A) and S. epidermidis ATCC 35984 (Fig. 2B) bacteria, respectively. The lower MIC measured for the MRSA-AH4807 strain is consistent with the approximately 2-fold higher fluorescence intensity of this strain upon incubation with CBD3-700DX compared to that of the S. epidermidis ATCC 35984 strain (Fig. 1B); this correlates with the higher number of CBD3-700DX molecules bound to each cell of the MRSA-AH4807 strain, as explained above. Importantly, no toxic effects were observed against either of the two staphylococcal species, neither in the presence of CBD3-700DX in the dark nor when they were exposed to red light at 30 J · cm^−2^ in the absence of the photosensitizer (Fig. 2A and B) (12).

Singlet oxygen (^1^O_2_) has been described as the predominant primary ROS involved in aPDT. However, photoactivation of IRDye 700DX will also lead to the production of other ROS, such as hydrogen peroxide (H_2_O_2_) (12). This is advantageous because H_2_O_2_ has a much longer half-life than ^1^O_2_ and can pass through biological membranes, thereby damaging different cellular compartments (27). Moreover, if generated in large quantities, H_2_O_2_ can act as a major precursor for highly reactive hydroxyl radicals (28). Therefore, we evaluated the production of H_2_O_2_ after aPDT with CBD3-700DX against MRSA-AH4807 using a dioxetane-based substrate which detects H_2_O_2_ and other peroxides in biological samples by luminescence. As shown in Fig. 2C, there was significantly higher production of H_2_O_2_ in the irradiated sample groups compared to the ones that were kept in the dark. Based on a H_2_O_2_ calibration curve (Fig. S1 in the supplemental material), we estimate that approximately 88 μM H_2_O_2_ can be produced after aPDT with 5 μM CBD3-700DX.

### CBD3-700DX disrupts *S. epidermidis* biofilms.

**FIG 3:**
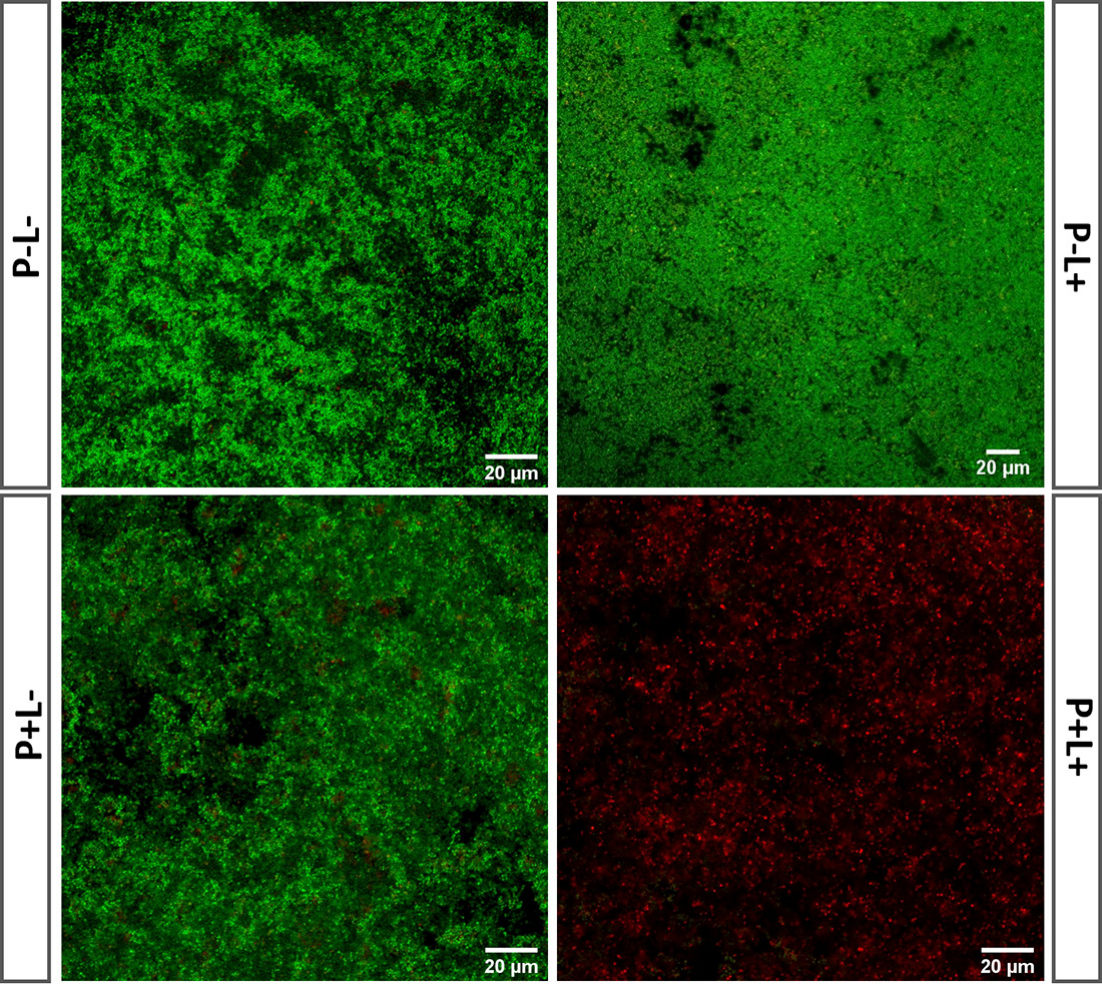
aPDT of S. epidermidis biofilms with CBD3-700DX. Biofilms formed by S. epidermidis ATCC strain 35984 were incubated with either 8 μM CBD3-700DX (*P*+) or PBS (*P*–), and they were either kept in the dark (*L*–) or treated with red-light LEDs at a radiance exposure of 30 J · cm^−2^ (*L*+). To assess bacterial viability, biofilms were stained with BacLight LIVE/DEAD stain and imaged by confocal laser scanning microscopy. Green fluorescence (Syto9) marks living bacteria and red fluorescence (propidium iodide) marks dead bacteria. Video S2 in the supplemental material shows a three-dimensional reconstruction from stacks of 2-dimensional confocal microscopy images recorded upon aPDT with CBD3-700DX (*P+L*+). Fig. S2 shows the unmerged images of the Syto9 and propidium iodide fluorescence.

To visualize the efficacy of CBD3-700DX toward staphylococcal biofilms, we grew biofilms of S. epidermidis on coverslips for 2 days, which were thereafter subjected to aPDT. In this respect, it should be mentioned that S. epidermidis is notorious for forming hard-to-eradicate biofilms on medical implants (29). Following the red-light exposure, live/dead staining was performed to mark viable and dead bacteria with the dyes Syto9 and propidium iodide, respectively. Fig. 3 and Fig. S2 show that 8 μM CBD3-700DX was not toxic to the biofilms when they were kept in the dark (*P+L*–), and neither was red-light irradiation with 30 J · cm^−2^ in the absence of CBD3-700DX (*P*–*L*+). Importantly, the combination of CBD3-700DX and red-light exposure (*P+L*+) leads to substantial disruption of the biofilm with massive bacterial killing. However, as demonstrated by the respective Z-stacks (Video S2), the main consequence of aPDT with CBD3-700DX is that it kills the bacteria in the upper layer of the biofilm and massively reduces its density, while leaving some viable bacteria in the deeper layers of the biofilm.

### (Photo)cytotoxicity of CBD3-700DX and intracellular ROS production.

**FIG 4:**
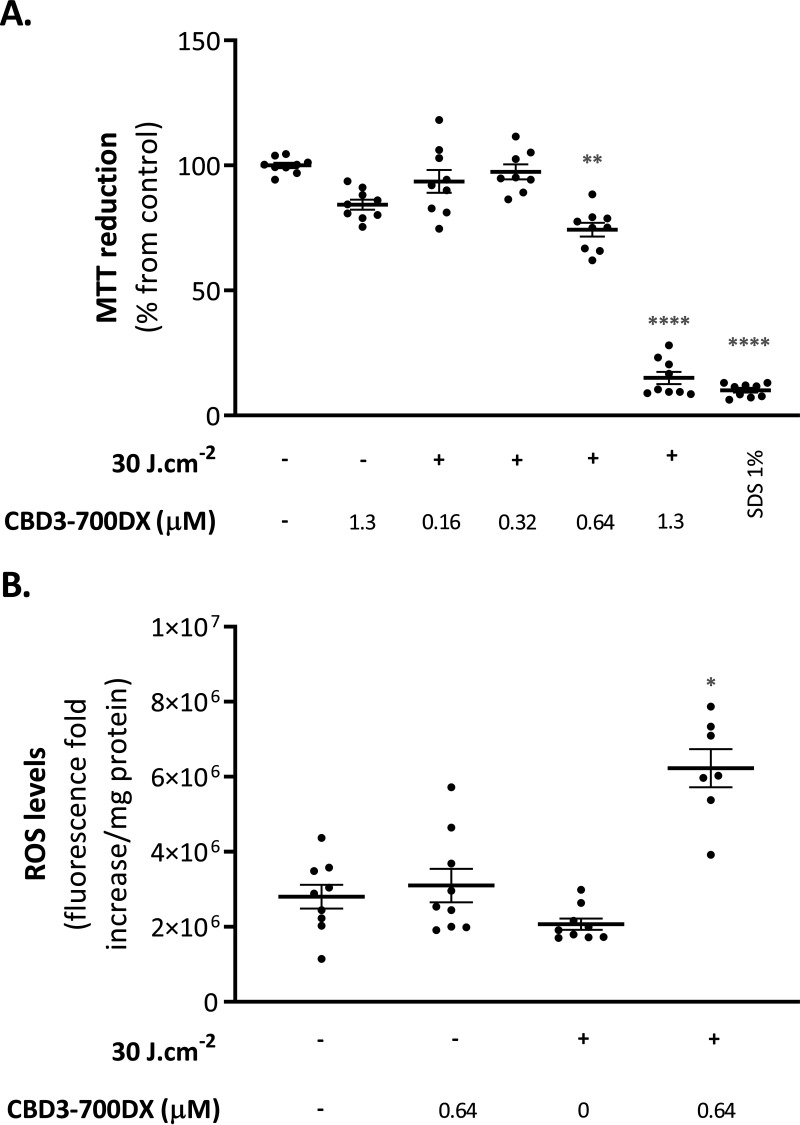
(Photo)cytotoxicity of CBD3-700DX toward HeLa cells. HeLa cells were incubated with different concentrations of CBD3-700DX for 15 min, and the unbound conjugate was removed by washing with DPBS prior to treatment with red light (+) at a radiance exposure of 30 J · cm^−2^. (A) (Photo)cytotoxicity was assessed using the colorimetric MTT assay 24 h after treatment. The percentage of cell viability, expressed as MTT reduction, was calculated relative to that of viable control cells that were mock-treated with DPBS in the dark. Cells treated with 1% SDS were used as a control for cell killing. (B) The H_2_DCFDA fluorescence fold increase per mg protein (*y* axis) was determined by fluorescence spectroscopy immediately after aPDT as a measure for cellular ROS levels. Data are presented as the mean ± SEM of three experiments performed in triplicate. Notably, in panel B, two outlier data points of 2.17 × 10^7^ and 3.90 × 10^7^ fluorescence fold increases per mg protein were removed from the condition where HeLa cells were treated with 0.64 μM CBD3-700DX in the presence of red light. An ordinary one-way ANOVA with a subsequent Holm-Sidak’s multiple-comparison test was used for statistical analysis. Significant differences compared with the control group (no photosensitizer and no light) are shown as follows: *, *P* < 0.03; **, *P* < 0.002; ***, *P* < 0.0002; ****, *P* < 0.0001.

To evaluate the (photo)cytotoxicity of CBD3-700DX toward mammalian cells, HeLa cells were incubated with different concentrations of CBD3-700DX. After 15 min incubation and washing off of unbound compound, the cells were either kept in the dark to assess cytotoxicity or irradiated with red light at 30 J · cm^−2^. Control experiments were performed where HeLa cells were mock-treated with Dulbecco’s phosphate-buffered saline (DPBS) or 1% SDS and kept in the dark. Cell viability was assessed 24 h after treatment using a colorimetric assay based on reduction of the yellow compound 3-(4,5-dimethylthiazol-2-yl)-2,5-diphenyltetrazolium Bromide (MTT) to a purple formazan precipitate by mitochondrial dehydrogenase activity (Fig. 4A). Thus, the reduction of MTT is directly correlated with the metabolic activity of living cells (30). Upon red-light exposure, there was evidence of mild photocytotoxicity at a CBD3-700DX concentration of 0.64 μM, whereas severe photocytotoxicity was observed at a concentration of 1.3 μM. Notably, at the latter CBD3-700DX concentration, only little cytotoxicity was observed in the dark. Since the observed photocytotoxicity could be attributed to insufficient washes of the unbound CBD3-700DX or to its internalization by HeLa cells, we performed two DPBS washes after exposure to the highest CBD3-700DX concentration used in this study (1.3 μM). Even though this resulted in a significant reduction in photocytotoxicity, the cellular viability was still reduced by more than 50% (Fig. S3). To verify if the conjugate was internalized by the HeLa cells, we determined the possible cytosolic production of ROS in HeLa cells treated with CBD3-700DX (Fig. 4B). After incubation with 0.64 μM CBD3-700DX and one wash with DPBS, cells were incubated with the intracellular ROS-sensitive probe 2′,7′-dichlorohydrofluorescin diacetate (H_2_DCFDA) immediately after red-light exposure (30 J · cm^−2^). A significant production of intracellular ROS was observed upon red-light exposure of the cells incubated with CBD3-700DX, indicating the internalization of CBD3-700DX in the HeLa cells (Fig. 4B). In contrast, no production of cytosolic ROS was observed in the cells that were incubated with the conjugate but kept in the dark.

### CBD3-700DX internalization and capture of intracellular *S. aureus* in HeLa cells.

**FIG 5:**
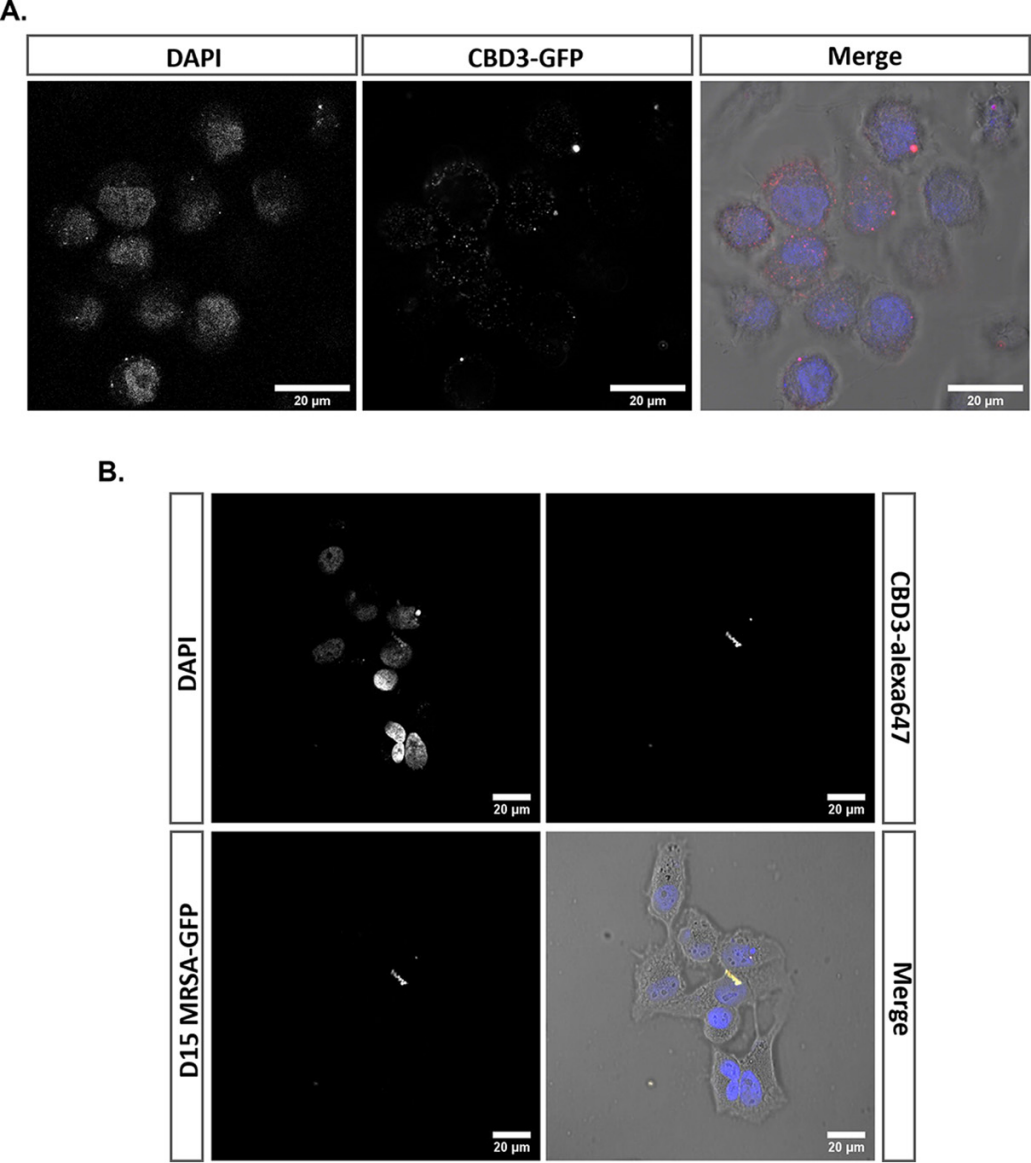
CBD3 internalization by HeLa cells and binding to intracellular S. aureus. (A) Uninfected HeLa cells incubated with 0.2 μM of the GFP-CBD3 fusion protein (false color represented in red). (B) HeLa cells were infected with CA-MRSA D15-GFP (MOI = 10) and subsequently incubated with lysostaphin to eliminate extracellular bacteria. The HeLa cells with internalized bacteria (bottom left image; green in the overlay) were then incubated with 0.5 μM CBD3-Alexa Fluor 647 (top right image; red in the overlay). Nuclei were stained with DAPI (top left image; blue in the overlay). Fluorescence images were acquired by confocal laser scanning microscopy. Note that in the overlay (bottom right image), the co-localizing fluorescence signals of the GFP (green) and CBD3-Alexa Fluor 647 (red) appear yellow.

The production of intracellular ROS prompted us to investigate the internalization of CBD3 by HeLa cells. Thus, confluent HeLa cells were incubated with GFP-CBD3 and then inspected by confocal fluorescence microscopy. The images presented in Fig. 5A show the internalization of GFP-CBD3 by HeLa cells and its possible localization around the cell nuclei, which were visualized by DAPI staining. To determine whether internalized CBD3 would bind to intracellular bacteria, we employed CBD3 conjugated to Alexa Fluor 647. Indeed, after elimination of extracellular bacteria with lysostaphin, the CBD3-Alexa Fluor 647 was shown to bind intracellular GFP-expressing bacteria of the CA-MRSA strain USA300 D15-GFP in infected HeLa cells (Fig. 5B; note the co-localization of GFP and CBD3-Alexa Fluor 647 fluorescence signals in the bottom right image). This demonstrates that CBD3 can serve as an agent to target and capture intracellular S. aureus.

### Killing of intracellular MRSA by aPDT with CBD3-700DX.

**FIG 6:**
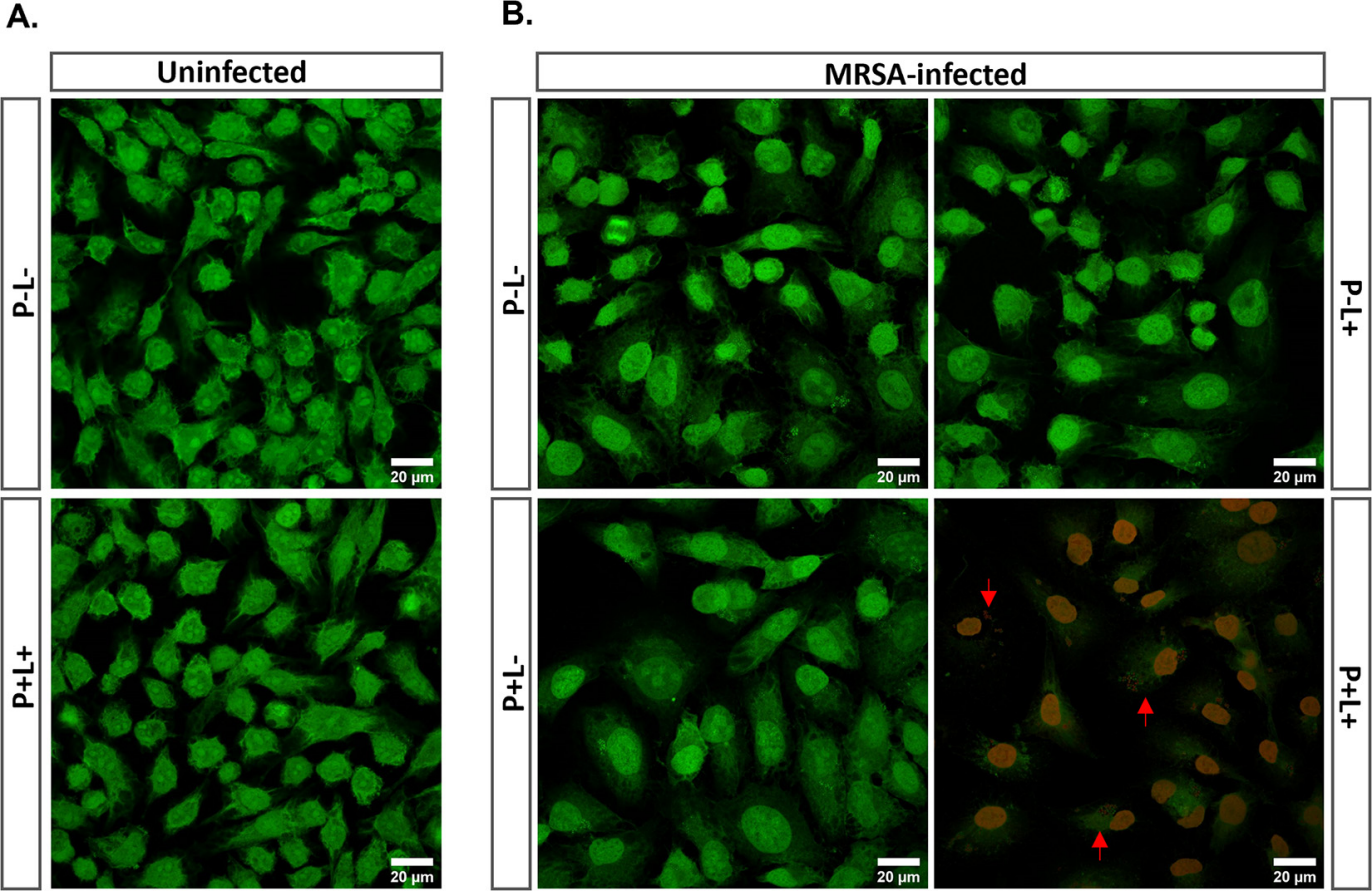
aPDT with CBD3-700DX kills intracellular S. aureus and infected HeLa cells. HeLa cells were incubated overnight without (*P*–) or with (*P*+) 0.2 μM CBD3-700DX. The following day, the cells were either infected with CA-MRSA-AH4807 at an MOI of 10 for 2 h, or they remained uninfected. Cells were then incubated with lysostaphin to eliminate extracellular bacteria. Both the uninfected (A) and the infected (B) HeLa cells were irradiated with red light (*L*+) at a radiance exposure of 30 J · cm^−2^ or were kept in the dark (*L*–). To assess bacterial and HeLa cell viability, BacLight LIVE/DEAD staining was performed followed by confocal laser scanning microscopy. Green fluorescence (Syto9) marks living cells and bacteria, while red fluorescence (propidium iodide) marks dead cells and bacteria (the red arrows mark dead bacteria). Fig. S4 and S5 in the supplemental material show unmerged images of the Syto9 and propidium iodide fluorescence for panels A and B, respectively.

After showing that CBD3-700DX can capture intracellular MRSA in HeLa cells, we investigated its ability to specifically kill this pathogen without off-target toxicity. Thus, HeLa cells were incubated overnight with 0.2 μM CBD3-700DX (a concentration which is not photocytotoxic for HeLa cells, Fig. 4A) and afterwards, these cells were infected with MRSA-AH4807. After 2 h infection and lysostaphin incubation to eliminate extracellular bacteria, cells were exposed to red light (30 J · cm^−2^) or kept in the dark. A control group of uninfected HeLa cells was included to investigate possible photocytotoxicity due to the overnight incubation with CBD3-700DX. Live/dead staining with Syto9 and propidium iodide was used to mark viable and dead cells, respectively. Notably, these two dyes will stain both bacterial and human DNA. As expected, no photocytotoxicity was observed in uninfected HeLa cells subjected to aPDT with CBD3-700DX (Fig. 6A and Fig. S4). Light alone (*P*–*L*+) or the presence of CBD3-700DX in the dark (*P+L*–) did not cause damage to the bacteria, nor to the infected mammalian cells. Importantly, however, the combination of CBD3-700DX with red light (*P+L*+) induced high intracellular bacterial killing (Fig. 6B and Fig. S5). Remarkably, we observed photocytotoxicity of CBD3-700DX toward the infected host cells only under this condition where intracellular bacteria were present.

## DISCUSSION

To contribute to the development of alternative strategies to fight persistent staphylococcal infections, we combined a bacterium-specific truncated staphylococcal endolysin (CBD3) (23) with a photoactivatable silicon phthalocyanine (IRDye 700DX) that has already made it to phase III clinical trials (31). The CBD moiety of endolysins provides high specificity and affinity in the nanomolar range to the peptidoglycan in bacterial cell walls, and it can even outperform the binding of endolysin-neutralizing antibodies, thereby overcoming possible host immune responses (32). Furthermore, CBD3 has been previously demonstrated to have high affinity for Staphylococcus species (23). Accordingly, in this study, we observed effective targeting of CBD3 toward S. aureus and S. epidermidis, and the killing of these staphylococcal species by red light-activated CBD3-700DX.

Even though the precise CBD3-binding site in the staphylococcal cell wall has not yet been determined, it seems most likely that the SH3b domain of CBD3 will bind to pentaglycine cross-bridges within the peptidoglycan, as this is typical for such domains (23). Consequently, we can not presently fully exclude the possibility of bacterial resistance to novel CBD3-based antimicrobial agents due to alterations in the muropeptide interpeptide cross-bridges, as has been previously reported for lysostaphin-resistant S. aureus isolates (33). Such alterations could potentially reduce the binding of CBD3 to staphylococcal cells, thereby reducing the efficacy of CBD3-based antimicrobials and aPDT agents. Nevertheless, pentaglycine cross-bridges are essential for S. aureus cells to withstand internal turgor, and the bacterial cells will not survive in their absence, which makes more drastic mutations unlikely (34). Also, there are no reports yet on bacterial resistance to aPDT; this is most likely due to the fact that ROS simultaneously damages multiple essential components of the bacterial cell, and to the fact that applied photosensitizers are only toxic upon light activation while the ROS produced at the targeted infection site are short-lived (8–10). Consequently, the infecting bacteria would have insufficient opportunity to develop resistance to aPDT.

S. epidermidis is one of the most common causes of primary bacteremia and infection of indwelling medical devices, due to its strong ability to colonize biomaterials by biofilm formation. Such biofilms are microbial aggregates in different cell physiologies, surrounded by biopolymeric substances. Consequently, the bacteria inside a biofilm are very resilient to host immune defenses and antimicrobial agents. It is, therefore, particularly noteworthy that targeted aPDT with CBD3-700DX causes massive destruction of S. epidermidis biofilm structure and kills the bacteria in the upper layer of the biofilm. In turn, this could potentially make the underlying biofilm-embedded bacteria more susceptible to antibiotic therapy and phagocytic killing.

To probe the possible clinical applicability of our aPDT approach based on CBD3-700DX, we investigated the cytotoxicity toward mammalian cells with or without red-light exposure. Our conjugate did not display cytotoxicity in the dark toward HeLa cells. Nevertheless, upon light activation, CBD3-700DX reduced cellular metabolism at concentrations higher than 0.3 μM. A higher number of washes reduced the observed cytotoxicity to some extent, but not completely, which probably relates to intracellular accumulation of CBD3-700DX. Consistent with this view, intracellular ROS production was observed for the aPDT-treated HeLa cells. Previous studies have shown that some endolysins may indeed become internalized by mammalian cells, thereby targeting “hidden” pathogens (32, 33, 35). Shen et al. (35) proposed a three-step process for this internalization based on membrane binding through electrostatic interaction and specific ligation, followed by cellular entry by endocytosis, and lastly by cytosolic transport to the pathogen of interest. Our present data show that not only does CBD3 become internalized by mammalian cells, but it can also specifically target intracellular S. aureus. Moreover, we show that this property of CBD3-700DX can be exploited to kill targeted intracellular S. aureus bacteria.

In our experimental setting, we preincubated HeLa cells overnight with the CBD3-700DX conjugate to show that subsequently invading bacteria can be captured. Indeed, the pre-internalized CBD3-700DX was found to bind the bacteria, allowing their elimination by red-light exposure. Interestingly, the viability of infected HeLa cells was reduced if they contained bacteria which were killed by aPDT with CBD3-700DX. In contrast, no such toxicity was observed for uninfected HeLa cells that had been incubated with CBD3-700DX overnight and were subsequently exposed to red light. These observations suggest an enhanced virulence of S. aureus upon intracellular exposure to ROS such as H_2_O_2_. This scenario is supported by the fact that the oxidative stress-responsive repressor of S. aureus, MgrA, regulates the production of toxins, such as alpha-toxin (36). These toxins might then damage the host cell membrane, leading to cell death. Thus, it is of interest for future studies to investigate whether particular toxins produced by the CA-MRSA-AH4807 strain in response to ROS exposure are indeed responsible for the observed HeLa cell killing. Alternatively, it is conceivable that the accumulation of dead bacteria or bacterial debris inside the host cell may trigger reactions which lead to apoptosis or necrosis.

In conclusion, the present study provides proof of principle that CBD3 can be applied as an effective targeting agent for anti-staphylococcal aPDT. It may thus serve to complement aPDT approaches which are based on antibodies that specifically target certain staphylococcal species (12). Although we did observe some photocytotoxicity of CBD3-700DX, it was remarkable to see this effect predominantly in mammalian cells with internalized bacteria. While this collateral damage to the infected cells may be perceived as a potential disadvantage, it can actually be advantageous for fully clearing an infection, since any intracellularly persisting bacteria will become exposed to the host’s innate and adaptive immune defences and to any co-administered antibiotics. To date, only two other approaches for targeted intracellular aPDT have been documented. In one approach, surface-bound hemoprotein receptors specific for S. aureus were targeted with gallium-substituted hemoglobin on silver nanoparticles (37). The other approach was based on peptide-chlorophyll conjugates that specifically target macrophages, allowing the subsequent light-induced killing of internalized S. aureus (38). We conclude that targeted aPDT with CBD3-700DX represents an attractive alternative to these two approaches because it allows the massive destruction of bacterial biofilms and is not restricted to bacteria internalized by professional phagocytes. However, it is important to bear in mind that aPDT with CBD3-700DX and any other photosensitizers will only be feasible for treatment of infections at sites where sufficient oxygen is available for the generation of ROS, and where the applied photosensitizer can be activated with red light. Taking these two prerequisites into account, aPDT will probably be mostly suitable for treating superficial infections or for intra-operative surgical debridement of infected implants and tissues.

## MATERIALS AND METHODS

### Production of CBD3, GFP-CBD3, and CBD3 conjugates.

CBD3 and GFP-CBD3 were produced as described in the literature (23). IRDye 700DX (LI-COR Biosciences, Nebraska, USA) and Alexa Fluor 647 dye (Thermo Fisher, Massachusetts, USA) were cross-linked to CBD3 via activated *N*-hydroxysuccinimide ester chemistry according to the manufacturer’s instructions.

### Visualization of CBD3 binding to MRSA and *S. epidermidis* by fluorescence microscopy.

The CA-MRSA-AH4807 (39) and S. epidermidis American Type Culture Collection (ATCC) 35984 strains were used in this study. The CA-MRSA strain AH4807 was derived from the CA-MRSA LAC strain AH126353, where the Photorhabdus luminescens *lux* operon was modified for Gram-positive bacterial expression and integrated at the Φ11 attachment site on a plasmid of the host (39). The different bacterial species were grown overnight in tryptic soy broth (TSB) in a shaking incubator at 37°C. Overnight cultures were diluted to an optical density measured at 600 nm (OD_600_) of 0.5. Cells were collected by centrifugation at 16,000 × *g* for 2 min and washed with phosphate-buffered saline (PBS). The washed cells were incubated with 1 μM GFP-CBD3 for 15 min in the dark, at room temperature (RT). After this, the cells were centrifuged again at 16,000 × *g* for 2 min and subsequently fixed in 4% paraformaldehyde (PFA) in PBS for 10 min. The bacterial DNA was stained with DAPI (Roche, Basel, Switzerland) followed by one PBS wash. Cells were spotted on a glass slide for microscopy. Image acquisition was performed with a Leica confocal laser scanning microscope (DMI6000, SP8, Wetzlar, Germany). The recorded images were processed using Image J software (National Institutes of Health, Bethesda, Maryland USA).

To quantify the binding of CBD3 to S. aureus or S. epidermidis, the same overnight cultures were diluted to an OD_600_ of 1. Next, cells from 1 mL of the diluted culture medium were collected by centrifugation at 16,000 × *g* for 2 min and washed with PBS. The cells were then incubated with 300 μL of 0.64 μM CBD3-700DX for 15 min in the dark, at RT. Planktonic bacteria were washed once with PBS to remove unbound CBD3-700DX and transferred to a black 96-well plate with a clear bottom for fluorescence imaging in an Amersham Typhoon Biomolecular Imager (Cytiva, Medemblik, the Netherlands). Fluorescence intensity values were analyzed with ImageJ. Each experiment was performed in triplicate at least twice.

### aPDT assay on planktonic Gram-positive bacteria.

The CA-MRSA-AH4807 and S. epidermidis ATCC 35984 strains were grown to exponential phase up to an OD_600_ of 0.5 in TSB, at 37°C, then harvested by centrifugation for 2 min at 16,000 × *g* and washed with PBS. The bacteria were subsequently 10-fold diluted, and 50-μL aliquots (∼1 × 10^7^ CFU/mL) were incubated with different concentrations of CBD3-700DX (0.02 to 2.6 μM) or PBS in a 96-well plate at RT for 15 min in the dark. They were then either kept in the dark or exposed to 30 J · cm^−2^ of red light. After the irradiation, bacteria were serially diluted in PBS, plated on blood-agar (BA) plates (5% sheep blood, Mediaproducts BV, Groningen, the Netherlands), and incubated aerobically for 16 h at 37°C for CFU counting. Each experiment was performed in triplicate, three times.

### H_2_O_2_ detection upon aPDT.

CA-MRSA-AH4807, grown as described above, was incubated with 5 μM CBD3-700DX and subjected to red light at a radiance exposure of 30 J · cm^−2^. H_2_O_2_ was detected with 10 μM of an AquaSpark Peroxide Probe (Biosynth, Staad, Switzerland), a dioxetane-based substrate for luminescent detection of H_2_O_2_ and other peroxides in biological samples. Quantification of bioluminescence was performed with a microplate spectrophotometer (Synergy HT, Biotek instruments, Vermont, USA). Each experiment was performed in triplicate, three times.

### Biofilm targeting by CBD3-700DX aPDT.

For biofilm formation, an S. epidermidis ATCC 35984 overnight culture was diluted to an OD_600_ of 0.1 in TSB supplemented with 5% glucose and 4% sodium chloride. Subsequently, 1-mL aliquots were transferred to 12-well plates containing 18-mm, chemically resistant, borosilicate glass coverslips. Upon 24 h of incubation at 37°C, planktonic bacteria on top of the coverslips were washed off with PBS. The biofilms were then incubated with CBD3-700DX (8 μM) for 15 min at RT. After washing with PBS to remove unbound immunoconjugate, biofilms were treated with red light at a radiance exposure of 30 J · cm^−2^ and stained with a LIVE/DEAD BacLight Bacterial Viability Kit (Thermo Fisher, Massachusetts, USA). Briefly, this kit contains two dyes: the Syto9 dye penetrates both viable and nonviable bacteria, while the propidium iodide penetrates bacteria with damaged membranes and quenches the fluorescence of Syto9. Live/dead-stained biofilms were examined using a Leica confocal laser scanning microscope (DMI 6000, SP8). The recorded images were processed using Image J software.

### Photo- and cytotoxicity of CBD3-700DX toward MRSA-infected human cells.

The human cervical cancer cell line HeLa (ATCC) was cultured in Dulbecco’s Modified Eagle’s (DMEM)-GlutaMAX medium (Gibco, New York, USA), supplemented with 10% fetal bovine serum, at 37°C and 5% CO_2_. Subsequently, 0.25% Trypsin-EDTA (Gibco) was used to detach adherent cells for subculturing. The detached cells were seeded into 96-well cell culture plates at a density of 3 × 10^4^ cells/well. On the following day, cells were treated with different concentrations of CBD3-700DX (0.16 to 1.3 μM) in the dark for 15 min. One group was washed with DPBS (1×) without calcium and magnesium (Lonza, Basel, Switzerland) and fresh DMEM medium was added, while the other group remained with the drug in DPBS. Irradiation with 30 J · cm^−2^ of red light was subsequently performed. Cell metabolic activity at 24 h post-aPDT was determined with an MTT assay, which measures the ability of HeLa cells to reduce MTT (Sigma-Aldrich, MO, USA) to colored formazan crystals. The formation of formazan was quantified with a microplate spectrophotometer (Synergy HT, Biotek Instruments) by measuring the absorbance at 570 nm, using 620 nm as the background wavelength. The percentage of absorbance for each treated sample was normalized to each untreated control. Each experiment was performed in triplicate, three times.

### Detection of intracellular ROS generation in human cells upon aPDT.

Immediately upon aPDT, HeLa cells were incubated with 5 μM H_2_DCFDA (Invitrogen, Massachusetts, USA), a chemically reduced form of fluorescein that can be used as an indicator for intracellular ROS generation, for 1 h at 37°C in the dark. After incubation with H_2_DCFDA, the cells were washed with DPBS, then mechanically scraped and resuspended in 1% (m/v) SDS solution in DPBS. Subsequently, the fluorescence was measured using a Synergy HT microtiter plate reader with excitation and emission filters of 485/20 and 540/25 nm. Protein concentrations were determined using the Pierce BCA Protein Assay Kit. Each experiment was performed in triplicate, three times.

### CBD3 internalization by human cells.

HeLa cells were seeded on coverslips in 24-well cell culture plates at a density of 2 × 10^5^ cells/well. After 24 h of incubation at 37°C and 5% CO_2_, HeLa cells were incubated with 0.2 μM GFP-CBD3, for 15 min in the dark. Cells were washed once with DPBS to remove unbound GFP-CBD3 and then fixed with PFA 4% for 10 min. Tween 0.5% was used to permeabilize the HeLa cells for 10 min. After one wash with DPBS, DAPI (Roche) was added to stain the cell nuclei for 5 min, followed by another wash. The coverslips were mounted on slides with Mowiol (Sigma-Aldrich) and left to dry overnight. Image acquisition was performed with a Leica confocal laser scanning microscope (DMI 6000, SP8). The recorded images were processed using ImageJ software (NIH).

### CBD3 binding to intracellular MRSA.

HeLa cells were seeded on coverslips in 24-well cell culture plates at a density of 2 × 10^5^ cells/well. After 24 h of incubation at 37°C and 5% CO_2_, HeLa cells were infected with S. aureus CA-USA300 D15-GFP (11) in exponential phase at a multiplicity of infection (MOI) of 30 for 2 h. Next, lysostaphin (AMBI Products, New York, USA, 25 μg · mL^−1^) was added to the infected cells for 30 min to eliminate all extracellular bacteria. CBD3, labeled with Alexa Fluor 647 according to the manufacturer’s manual (0.5 μM), or DPBS was then added to the infected cells, and incubation was continued for 15 min in the dark. The HeLa cells were washed once with DPBS to remove the unbound CBD3-Alexa Fluor 647 and then fixed with PFA 4% for 10 min. Tween 0.5% was used to permeabilize the HeLa cells for 10 min. After one wash with DPBS, DAPI was added to stain the cell nuclei for 5 min, followed by another wash. The coverslips were mounted on slides with Mowiol and left to dry overnight. Image acquisition was performed with a Leica confocal laser scanning microscope (DMI 6000, SP8). The recorded images were processed using ImageJ software (NIH).

### Selective killing of intracellular MRSA by CBD3-700DX.

HeLa cells were seeded on coverslips in 24-well cell culture plates at a density of 2 × 10^5^ cells/well, together with 0.2 μM CBD3-700DX. After 24 h of incubation at 37°C and 5% CO_2_, the HeLa cells were washed once with DPBS and then infected with CA-MRSA-AH4807 in exponential phase at an MOI of 15 for 1 h. Lysostaphin (25 μg · mL^−1^) was added to the infected cells for 30 min to eliminate all extracellular bacteria. The HeLa cells were washed once with DPBS and DMEM was added to the wells before irradiation with red light at a radiance exposure of 30 J · cm^−2^; or, alternatively, the cells were kept in the dark. Lastly, the cells were stained with the LIVE/DEAD BacLight Bacterial Viability Kit (Thermo Fisher). The coverslips with live/dead-stained cells were mounted on slides with Mowiol and left to dry overnight. Image acquisition was performed with a Leica confocal laser scanning microscope (DMI 6000, SP8). The recorded images were processed using ImageJ software (NIH).

### Statistics.

The results are presented as the mean ± standard error of the mean (SEM). All statistical analyses were performed with GraphPad Prism version 8.0.1. Statistical significance of differences among two unpaired groups was evaluated with the Welch’s test. Variables in three or more unmatched groups were assessed with Kruskal-Wallis tests, and subsequently by the Dunn’s or Dunnett’s multiple-comparison tests, or by ordinary one-way analysis of variance (ANOVA) with a subsequent Holm-Sidak’s multiple-comparison test, depending on the Gaussian distribution of residues. *P* values of <0.05 were considered significant.
