# Supplementary material for: Targeted Antimicrobial Photodynamic Therapy of Biofilm-Embedded and Intracellular Staphylococci with a Phage Endolysin’s Cell Binding Domain
Source: Microbiol Spectr. 2022 Feb 23;10(1):e01466-21. doi: 10.1128/spectrum.01466-21 (PMC8865409; doi:10.1128/spectrum.01466-21)
Supplement: SUPPLEMENTAL FILE 1 — Supplemental material. Download SPECTRUM01466-21_Supp_1_seq8.pdf, PDF file, 3.6 MB [file spectrum01466-21_supp_1_seq8.pdf]

Supplementary Figures

Targeted antimicrobial photodynamic therapy of biofilm-embedded and intracellular staphylococci with a phage endolysin's cell binding domain

Mafalda Bispo[a], Sílvio B. Santos[b], Luís D. R. Melo[b], Joana Azeredo[b], Jan Maarten van Dijl[a]#

[a]Department of Medical Microbiology, University of Groningen, University Medical Center Groningen, Groningen, the Netherlands.

[b]Centre of Biological Engineering, University of Minho, Campus de Gualtar, Braga, Portugal

Running title: Targeted aPDT with a phage endolysin domain

#Address correspondence to Jan Maarten van Dijl, j.m.van.dijl01@umcg.nl

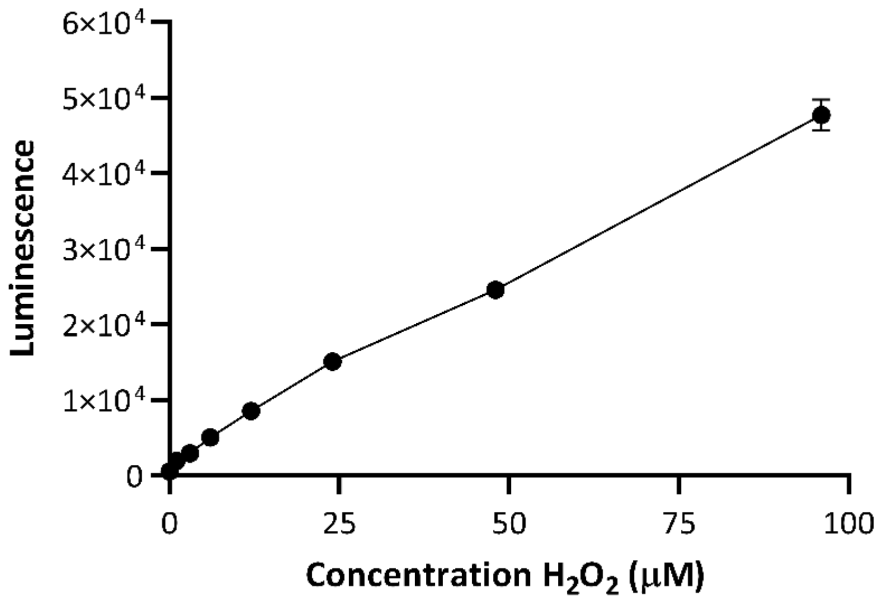

Figure S1. Calibration curve for H_2O_2 detection with 10 μM of an AquaSpark Peroxide Probe.

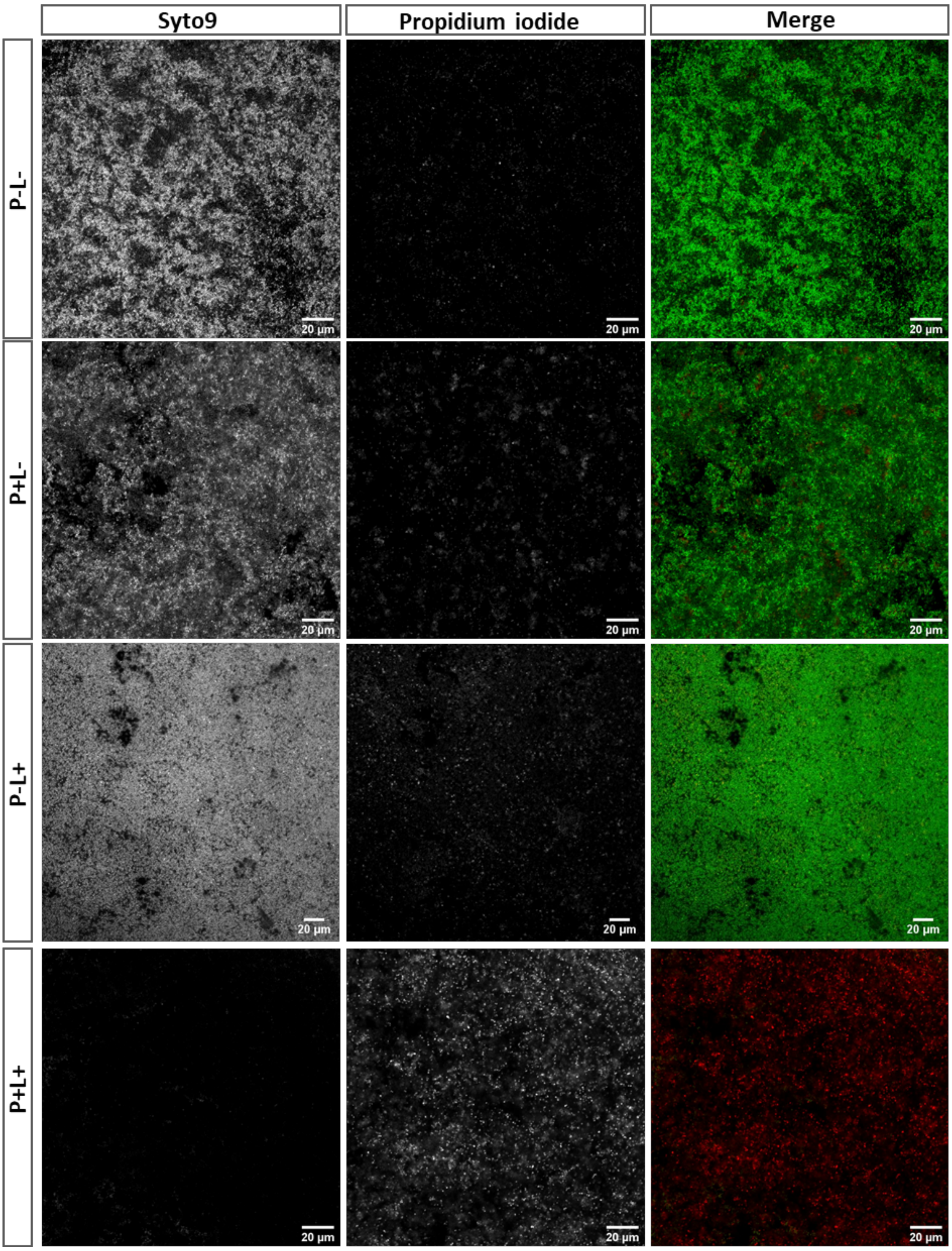

Figure S2. aPDT of *S. epidermidis* biofilms with CBD3-700DX. Biofilms formed by *S. epidermidis* ATCC strain 35984 were either incubated with 8 µM of CBD3-700DX (P+) or PBS (P-), and they were either kept in the dark (L-) or treated with red light LEDs at a radiant exposure of 30 $J.cm^{-2}$ (L+). To assess the bacterial viability, biofilms were stained with the BacLight LIVE/DEAD stain and imaged by confocal laser scanning microscopy. Green fluorescence (Syto9) marks living bacteria, and red fluorescence (propidium iodide) marks dead bacteria. Supplementary movie S1 shows a three-dimensional video representing the inside of the P+L+ biofilm. Supplemental Figure S2 shows the unmerged images of the Syto9 and propidium iodide fluorescence. The merged images are presented in Figure 3 of the main manuscript.

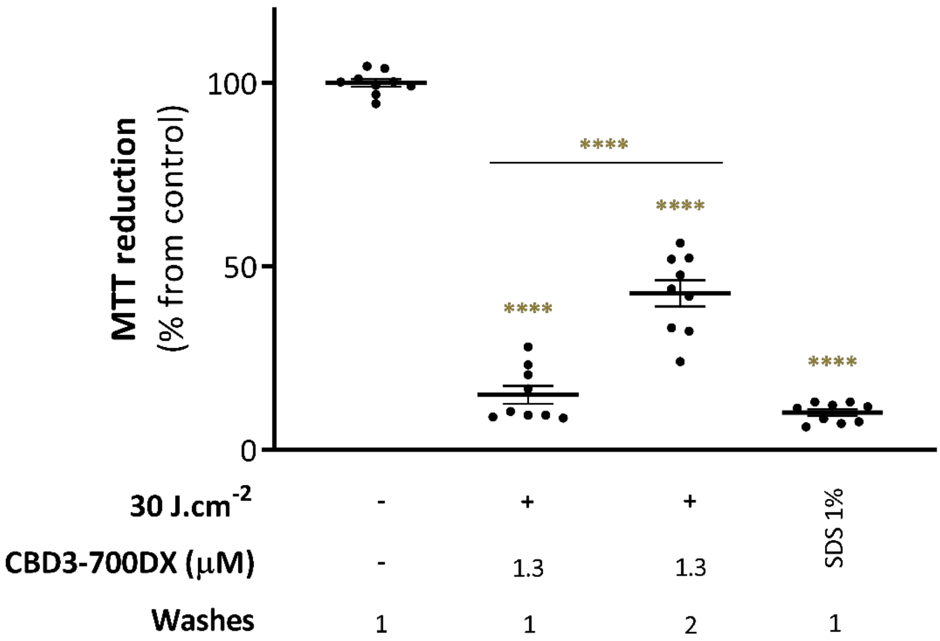

Figure S3. Impact of the number of washes on the (photo-)cytotoxicity of CBD3-700DX towards HeLa cells. HeLa cells were incubated with CBD3-700DX for 15 min, and the unbound conjugate was removed by washing once or twice with DPBS prior treatment with red light (+) at a radiant exposure of 30 J.cm^{-2}. (Photo-)cytotoxicity was assessed using the colorimetric MTT assay 24 h after treatment. The percentage of cell viability expressed as MTT reduction was calculated relative to viable control cells that were mock-treated with DPBS in the dark. Cells treated with 1 % SDS were used as a control for cell killing. Data are presented as the mean ± SEM of three experiments performed in triplicates. An ordinary one-way ANOVA with a subsequent Holm-Sidak's multiple-comparison test was used for statistical analysis. Significant differences compared with the control group (no photosensitizer and no light) are marked as follows: *P < 0.03**P < 0.002; ***P < 0.0002; ****P < 0.0001.

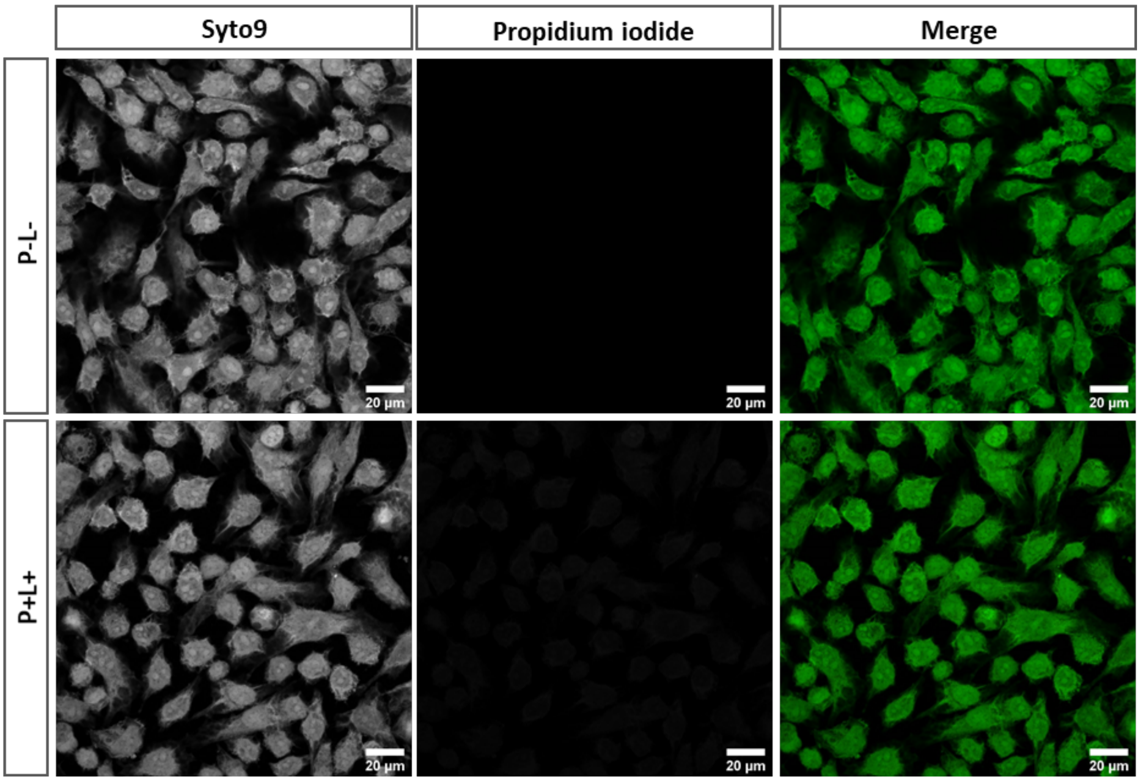

Figure S4. Overnight treatment with CBD3-700DX followed by aPDT is not toxic to HeLa cells. HeLa cells were incubated overnight without (P-) or with (P+) 0.2 µM of CBD3-700DX. The following day, the HeLa cells were irradiated with red light (L+) at 30 J.cm^{-2} or kept in the dark (L-). To assess the HeLa cell viability, BacLight LIVE/DEAD staining was performed, followed by confocal laser scanning microscopy. Green fluorescence (Syto9) marks living cells. Red fluorescence (propidium iodide) would mark dead bacteria but remained undetected. The merged images are presented in Figure 6A of the main manuscript.

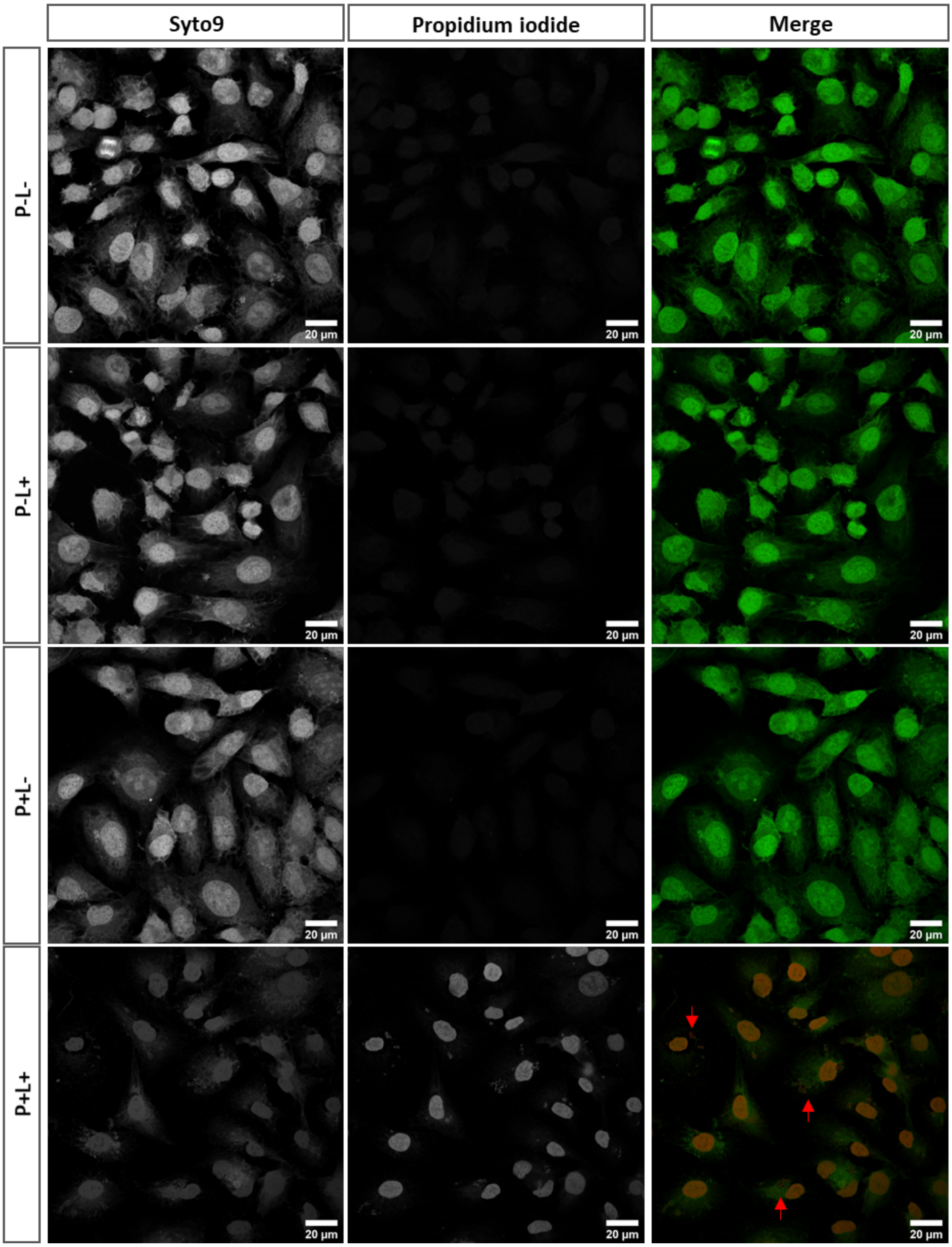

Figure S5. aPDT with CBD3-700DX kills intracellular *S. aureus* and infected HeLa cells. HeLa cells were incubated overnight without (P-) or with (P+) 0.2 µM of CBD3-700DX. The following day, the cells were infected with CA-MRSA D15-GFP at a MOI of 10 for 2 h. The infected cells were then incubated with lysostaphin to eliminate extracellular bacteria, and irradiated with red light (L+) at a radiant exposure of 30 $J.cm^{-2}$ or kept in the dark (L-). To assess the bacterial and HeLa cell viability, BacLight LIVE/DEAD staining was performed, followed by confocal laser scanning microscopy. Green fluorescence (Syto9) marks living cells and bacteria, while red fluorescence (propidium iodide) marks dead cells and bacteria (the red arrows mark dead bacteria). The merged images are presented in Figure 6B of the main manuscript.

Video S1. Three-dimensional reconstruction from stacks of two-dimensional confocal microscopy images of a *S. epidermidis* ATCC strain 35984 biofilm stained with the BacLight Live/Dead stain. The biofilm was incubated with 8 µM of CBD3-700DX and treated with red light LEDs at a radiant exposure of 30 $J.cm^{-2}$. Green fluorescence (Syto9) marks living bacteria, and red fluorescence (propidium iodide) marks dead bacteria. Scale bar: 20 µm. The video corresponds to the P+L+ biofilm image in Figure 3 of the main manuscript.
